# RIPK3 Promotes JEV Replication in Neurons *via* Downregulation of IFI44L

**DOI:** 10.3389/fmicb.2020.00368

**Published:** 2020-03-24

**Authors:** Peiyu Bian, Chuantao Ye, Xuyang Zheng, Chuanyu Luo, Jiali Yang, Mengyuan Li, Yuan Wang, Jing Yang, Yun Zhou, Fanglin Zhang, Jianqi Lian, Ying Zhang, Zhansheng Jia, Yingfeng Lei

**Affiliations:** ^1^Department of Infectious Diseases, Tangdu Hospital, Air Force Medical University, Xi’an, China; ^2^Pathogenic Biology, Medical College of Yan’an University, Yan’an, China; ^3^Key Laboratory of Resource Biology and Biotechnology in Western China, Ministry of Education, College of Life Sciences, Northwest University, Xi’an, China; ^4^Department of Microbiology, School of Preclinical Medicine, Air Force Medical University, Xi’an, China

**Keywords:** Japanese encephalitis virus (JEV), receptor interacting serine/threonine-protein kinase 3 (RIPK3), interferon-induced protein 44-like gene (IFI44L), neurons, cellular innate immune response

## Abstract

Japanese encephalitis virus (JEV), the leading cause of viral encephalitis in Asia, is neurovirulent and neuroinvasive. Neurons are the main target of JEV infection and propagation. Receptor interacting serine/threonine-protein kinase 3 (RIPK3) has been reported to contribute to neuroinflammation and neuronal death in many central nervous system diseases. In this study, we found that the progression of JE was alleviated in RIPK3-knockout (RIPK3^–/–^) mice in both peripheral and intracerebral infection. RIPK3-knockdown (RIPK3-RNAi) neuro2a cells showed higher cell viability during JEV infection. Moreover, the JEV load was significantly decreased in RIPK3^–/–^ mouse-derived primary neurons and RIPK3-RNAi neuro2a cells compared with wild-type neurons, but this was not observed in microglia. Furthermore, RNA sequencing of brain tissues showed that the level of the interferon (IFN)-induced protein 44-like gene (IFI44L) was significantly increased in JEV-infected RIPK3^–/–^ mouse brains, RIPK3^–/–^ neurons, and RIPK3-RNAi-neuro2a cells. Then, it was demonstrated that the propagation of JEV was inhibited in IFI44L-overexpressing neuro2a cells and enhanced in IFI44L and RIPK3 double knockdown neuro2a cells. Taken together, our results showed that the increased expression of RIPK3 following JEV infection played complicated roles. On the one hand, RIPK3 participated in neuroinflammation and neuronal death during JEV infection. On the other hand, RIPK3 inhibited the expression of IFI44L to some extent, leading to the propagation of JEV in neurons, which might be a strategy for JEV to evade the cellular innate immune response.

## Introduction

Japanese encephalitis virus (JEV) is a positive-sense, single-stranded RNA virus belonging to the genus *Flavivirus* in the family *Flaviviridae*. JEV is both neurovirulent and neuroinvasive and can lead to severe encephalitis (Lannes et al., 2017). Glycoprotein E mediates JEV entry through attachment and endocytosis, followed by membrane fusion and uncoating (Wang et al., 2017; Yun and Lee, 2018). Pattern recognition receptors (PRRs), such as retinoic acid-inducible gene 1-like receptors (RIG-I) and Toll-like receptor 3 (TLR3), in infected cells can recognize viral components and induce the production of interferons (IFNs), which then drive the expression of various IFN-stimulated genes (ISGs) through the IFN receptor (IFNR)/Janus kinase (Jak1)/tyrosine kinase (Tyk)2/signal transducer and activator of transcription (STAT)1/STAT2 pathway to fight against virus invasion (Liu et al., 2013; Han et al., 2014). As a result of such interactions, JEV has developed many strategies to counteract the host innate immune response (Ye et al., 2017; Zhou et al., 2018).

Receptor interacting serine/threonine-protein kinase 3 (RIPK3) has been shown to participate in several biological or pathological processes and play complicated and even controversial roles in different host cells during various viral infections (He and Wang, 2018). The activation of RIPK3 and subsequent mixed lineage kinase domain-like pseudokinase (MLKL) phosphorylation can lead to cellular necroptosis and damage-associated molecular pattern (DAMP) production (Pasparakis and Vandenabeele, 2015). It has been reported that RIPK3-mediated necroptosis destroys host cells and limits the propagation of viruses such as herpes simplex virus (HSV), influenza virus (IAV), and vaccinia virus (VV) (Wang et al., 2014; Huang et al., 2015; Nogusa et al., 2016; Koehler et al., 2017). RIPK3 also promoted or inhibited the propagation of virus in a cell death-independent manner during coxsackievirus B3 (CVB), IAV, and Zika virus (ZIKV) infections (Harris et al., 2015; Downey et al., 2017; Daniels et al., 2019). Additionally, it has been reported that RIPK3 contributes to the production of chemokines CXCL10 and CCL2 in West Nile virus (WNV)-infected neurons to recruit T lymphocytes and inflammatory myeloid cells to the central nervous system (CNS) (Daniels et al., 2017). In a previous study, we found that JEV infection induced the expression of MLKL, leading to necroptosis of neurons and neuroinflammation, which was shown to be alleviated in JEV-infected MLKL-knockout mice (Bian et al., 2017). However, the role of RIPK3 in JEV infection is unknown.

In this study, we found that the survival rate of RIPK3-knockout (RIPK3^–/–^) mice was significantly increased after JEV infection compared to that of wild-type (WT) mice. The expression of RIPK3 in neurons was increased after JEV infection, and cell viability was improved after RIPK3 knockdown. We also found that the replication of JEV in RIPK3^–/–^ mice and neurons was inhibited to some extent. Comparison of the RNA-sequencing results in JEV-infected brain tissues between WT and RIPK3^–/–^ mice showed that a series of IFN-stimulated genes (ISGs) were upregulated in RIPK3^–/–^ mice, especially the IFN-induced protein 44-like gene (IFI44L). Then, it was demonstrated that IFI44L inhibited JEV propagation in neuronal cells, and the increased expression of IFI44L contributed to the inhibition of JEV in RIPK3^–/–^ neuronal cells. Thus, we speculated that the slightly increased RIPK3 might be a strategy for JEV to evade cellular immunity in neurons.

## Materials and Methods

### Ethics Statement

All animal experiments were reviewed and approved by the Animal Care and Use Committee of the Laboratory Animal Center, Air Force Medical University. The number of Animal Experimental Ethical Inspection is 20160112. And all experiments were carried out complying with the recommendations in the Guide for the Care and Use of Laboratory Animals.

### Receptor Interacting Serine/Threonine-Protein Kinase 3-Knockout Mice

The RIPK3^±^ C57BL/6 mice were a gift from the lab of Dr. Yazhou Wang (Department of Neurobiology and Collaborative Innovation Center for Brain Science, School of Basic Medicine, Air Force Medical University) and were kept in a specific pathogen-free (SPF) facility. Toe DNA from newborn mice was extracted and amplified with PrimeStar (Takara, Japan). Then, the products were analyzed by agarose gel electrophoresis to screen WT, RIPK3^±^, RIPK3^–/–^ descendants. WT and RIPK3^–/–^ mice (6–8 weeks) were infected with 5 × 10^6^ JEV plaque-forming units (PFUs) in 20 μl phosphate-buffered saline (PBS) by footpad injection or 100 PFU in 2 μl *via* intracerebral injection. The weight, behavior score, and death cases of each group were recorded twice a day at 8:00–9:00 and 16:00–17:00 for 20 days until all the groups were totally stable. The scoring criteria were as follows: 0: no significant abnormal behaviors, piloerection, restriction of movement, body stiffening, or hind limb paralysis; 1: piloerection, no restriction of movement, body stiffening, or hind limb paralysis; 2: piloerection, restriction of movement, no body stiffening or hind limb paralysis; 3: piloerection, restriction of movement, body stiffening, no hind limb paralysis; 4: piloerection, restriction of movement, body stiffening, and hind limb paralysis; 5: piloerection, restriction of movement, body stiffening, hind limb paralysis, sometimes tremor and even death.

### Cells and Virus

The JEV-P3 strain was propagated in the brains of 3-day-old inbred BALB/C suckling mice and titrated by conventional plaque assay.

The neuroblast cell line Neuro2a, baby hamster kidney (BHK) cells, and HEK 293T cells [purchased from American Type Culture Collection (ATCC)] were cultured in Dulbecco’s modified Eagle’s medium (DMEM; Gibco, Grand Island, NY, United States) containing 10% fetal bovine serum (FBS; Gibco, Grand Island, NY, United States) and 1% penicillin streptomycin combination (PS). The microglia cell line N9 (purchased from ATCC) was cultured in DMEM with 5% FBS and 1% PS.

### Immunohistochemical Staining

Mice were administered propidium iodide (PI; 4 mg/ml, Sigma, in 0.9% NaCl) intraperitoneally (100 μl/20 g weight) and euthanized 1 h later. Brains were harvested and protected from light. Brain sections of 10 μm were prepared with a vibratome. The slides were incubated with primary anti-RIPK3 antibody (Abcam, Cambridge, MA, United States) in PBS containing 0.1% Triton X-100 and 1% bovine serum albumin (BSA) at 4°C for 16 h. After washing, the sections were incubated with the secondary antibodies for 1 h at room temperature. The nuclei were counterstained with 4′,6-diamidino-2-phenylindole (DAPI), and coverslips were placed on the samples with 50% glycerol in PBS.

### RNA Sequencing Analysis

Wild-type and RIPK3^–/–^ mice (4–6 weeks) were injected intracerebrally with PBS or 100 PFU JEV in 2 μl. Brains were harvested at 3 days post infection (dpi) and washed with 4°C PBS three times and then stored in liquid nitrogen. Then, the total RNA was extracted for RNA sequencing. The expression values [reads per kilobase million (RPKM)] were normalized per gene over all samples, the mean and standard deviation (SD) of expression over all samples were calculated for each gene, and the expression value was linearly transformed using the formula (RPKM-mean)/SD. The results were analyzed using the Dr. Tom network platform of BGI^1^ and GraphPad Prism 7.

### DNA Construction

To inhibit the expression of RIPK3, the shRNA targeting mouse RIPK3 (5′-GCTGGAGTTTGTGGGTAAAGG-3′) was constructed. The mouse IFI44L gene segment with sites for the restriction endonucleases *Bgl*II and *Mlu*I was generated through PCR (primer sequence in Supplementary Table S1) with Q5 High-Fidelity DNA Polymerase (NEB, United States) and cloned into the Lenti-GFP-zeocin plasmid (pLenti-GZ) (*via Bam*HI and *Mlu*I restriction digests). The plasmids from positive clones were extracted and sequenced. Then, the recombinant IFI44L overexpression plasmid was obtained. Three oligos targeting IFI44L (sequences in Supplementary Table S1) with the restriction endonucleases *Age*I and *Eco*RI were annealed and inserted into the pLK0.1-puro plasmid. The plasmids from positive clones were extracted and sequenced to obtain the correct recombinant interfering plasmid.

### Generation and Purification of Recombinant Lentiviral Particles

Lentiviral pseudoparticles were generated by cotransfecting 293T cells in T75 flasks with the plasmids pLenti-IFI44L-GFP-zeocin, pLenti-shRNAi-RIPK3-puro, or pLenti-shRNAi-IFI44L-puro (1, 2, and 3) proviral DNA (12 μg); envelope plasmid (pMD2. G, 6 μg); and packing plasmid (psPAX2, 9 μg). Before transfection, 9 ml DMEM was added to each T75 flask. For each transfection, 108 μl transfection regent LipoFectMAX (ABP Biosciences, United States) was mixed with 27 μg total DNA in 2 ml DMEM for 30 min and then added to the T75 flask. The cells were maintained at 37°C for 6 h, after which the medium was changed to DMEM with 2% FBS. The supernatants were harvested at 48 and 72 h. The cell debris was removed by centrifugation at 1,000 × *g* for 10 min and then 10,000 × *g* for 35 min. Subsequently, the viral suspension was concentrated at 165,000 × *g* for 4 h at 4°C, and the virus particles were harvested in 500 μl DMEM and stored at −80°C.

### Lentivirus Infection and Positive Cell Screening

Neuro2a cells and N9 cells were seeded into six-well plates at 4 × 10^5^ overnight. The supernatant was removed, and RIPK3-shRNA lentiviral particles mixed with polybrene (1 μg/ml) were added. After infection for 4 h, 1 ml DMEM with 10% FBS was added. Then, 48 h later, DMEM containing puromycin was added to neuro2a cells (2 μg/ml) and N9 cells (10 μg/ml) to screen the positive cells. Neuro2a cells with IFI44L overexpression or downregulation by shRNA were also constructed as described above.

### Plasmid Transfection

Neuro2a cells and RIPK3-RNAi neuro2a cells were plated in six-well plates at 4 × 10^5^ overnight. The supernatant was discarded, and the mixture of pCMV-GFPSpark or pCMV-RIPK3-OFPSpark (Sino Biological, China) (2 μg) with LipoFectMAX (6 μl) in 1 ml DMEM was added to each well. After incubation for 6 h, the medium was changed to 10% FBS-containing DMEM. Then, 24 h after transduction, the cells were infected with JEV-p3 at a multiplicity of infection (MOI) of 0.1. At 12 and 24 h post infection (hpi), cells and supernatant were harvested for qRT-PCR and conventional plaque assay.

### Cellular Viability Assay

Neuro2a cells were inoculated into opaque-walled 96-well plates at 10,000/well and maintained overnight. After JEV infection, viability was tested with a CellTiter-Glo Assay kit (Promega, United States). According to the protocol, the substrate and buffer were mixed thoroughly to obtain the detection reagent, and the plates were equilibrated at room temperature for approximately 30 min before the experiments. Then, 100 μl of detection reagent was added to the plates containing 100 μl of medium and mixed on an orbital shaker for 2 min to induce cell lysis. Then, the plates were incubated at room temperature for 10 min. The luminescence signal was recorded with a Bio-Tek Synergy HT Multi-Detection Microplate Reader and analyzed with GraphPad Prism 7.

### Isolation and Culture of Primary Neurons

Mice pregnant for 16–17 days were sacrificed, and the embryos were excised. The embryonic brains were harvested, and the meninges were removed completely. Then, the cerebral cortices were dissected and treated with papain (2 mg/ml) for 15 min, and 2 ml FBS was added to terminate the digestion. The liquid was removed, and the tissues were gently dissociated in DMEM with 10% FBS by pipetting. Then, the tissue suspension was filtered through a 70-μm cell strainer (Falcon, BD, United States). The isolated cells were seeded onto poly-L-lysine (100 μg/ml; Sigma, United States)-coated 60-mm plates and cultured in a humidified atmosphere at 37°C. After 24 h, the medium was changed to serum-free neurobasal medium (Gibco, United States) containing B27 (Gibco, United States) and L-glutamine (Gibco, United States).

### Virus Infection

Neuro2a cells or modified neuro2a cells were seeded in six-well or 96-well plates at a density of 2 × 10^5^/well or 10,000/well overnight. Then, the cells were infected with JEV (MOI = 0.1). After incubation for 1 h, the virus suspension was removed, and fresh DMEM was added. The cells and supernatant were harvested at different time points (24, 48, 72 h after infection) for qRT-PCR, Western blotting (WB), and conventional plaque assay.

N9 cells and RIPK3-shRNA N9 cells were seeded in six-well plates at a density of 4 × 10^5^/well overnight. Then, the cells were infected with JEV (MOI = 1). After incubation for 1 h, the virus suspension was removed, and fresh DMEM was added. The cells and supernatant were harvested at different time points (24, 48, 72 h after infection) for qRT-PCR, WB, and conventional plaque assay.

### qRT- PCR

WT and RIPK3^–/–^ mice were euthanized and perfused with PBS, and then the whole brain of each mouse was harvested and stored at −80°C. Total RNA from mouse brains and cells was extracted with RNAfast1000 (PIONEER, China). cDNA was prepared by reverse transcription with total RNA as the template using the PrimeScript RT reagent Kit (TaKaRa, Japan). qRT-PCR experiments were carried out using SYBR Green Real-Time PCR Master Mix (TaKaRa, Japan) according to the manufacturer’s instructions (for the primers used in this study, see Supplementary Table S1). The mRNA expression was normalized to β-actin expression, and the data are shown as the relative change to the corresponding reference for each group.

### Western Blotting

Total protein from the brain of each mouse or from cells was extracted with radioimmunoprecipitation assay (RIPA) buffer containing phenylmethanesulfonyl fluoride (PMSF) and phosphatase inhibitors and then quantified using a Protein Reagent Assay BCA Kit (Thermo, Waltham, MA, United States). Thirty micrograms of protein from each sample was loaded and electrophoresed using 12% sodium dodecyl sulfate-polyacrylamide gel electrophoresis (SDS-PAGE) gels and then transferred onto polyvinylidene difluoride (PVDF) membranes (Millipore, Billerica, MA, United States). After being blocked with 3% BSA at room temperature for 60 min, the membranes were incubated with primary antibodies (see Supplementary Table S1) overnight at 4°C. Then, the blots were incubated with the corresponding DyLight 800/700-labeled secondary antibodies for 2 h at room temperature. The blots were visualized using an infrared imaging system (Odyssey, LI-COR, Lincoln, NE, United States).

### Plaque Assay

BHK cells were seeded in six-well plates at 4 × 10^5^/well overnight. The supernatant of the cells was removed, and the cells were washed with 1 × PBS twice. Then, serial 10-fold diluted samples with DMEM were added and incubated at 37°C for 2 h. The viral supernatant was replaced with 4 ml overlay media (25 ml 4 × DMEM, 50 ml 4% methylcellulose, 2 ml FBS, 23 ml ddH_2_O) for 5 days. The overlay medium was washed off with 1 × PBS, and the cells were fixed with 4% paraformaldehyde (PFA) for 30 min. Crystal violet dye was added at 2 ml per well for 15 min and washed off with running tap water. Finally, the plaques were counted.

### Statistical Analysis

All statistical analyses were performed using GraphPad Prism version 7.01 software. Statistical differences were determined using Student’s *t*-test or two-way analysis of variance (ANOVA). *P*-values < 0.05 were considered significant.

## Results

### Receptor Interacting Serine/Threonine-Protein Kinase 3-Knockout Mice Showed Decreased Morbidity and Mortality After Japanese Encephalitis Virus Infection

**FIGURE 1:**
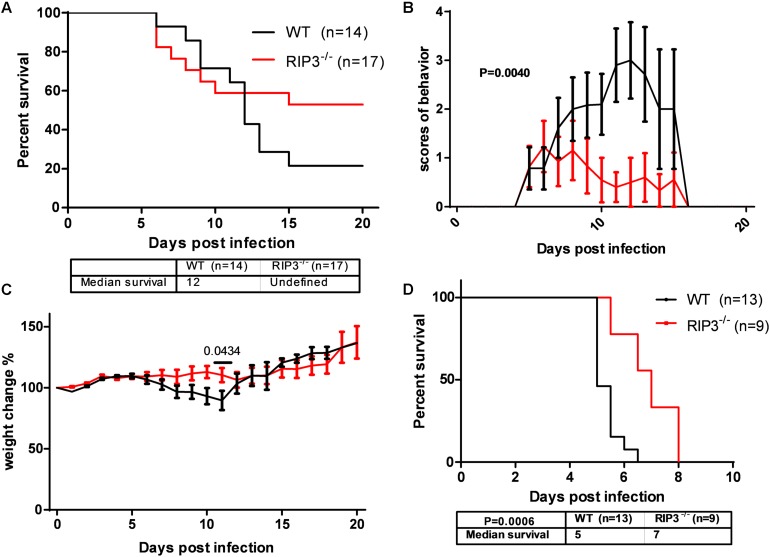
Receptor interacting serine/threonine-protein kinase 3 (RIPK3)-knockout mice showed decreased morbidity and mortality after Japanese encephalitis virus (JEV) infection *via* peripheral and intracerebral infection. **(A)** RIPK3^–/–^ (*n* = 17) and wild-type (WT; *n* = 14) C57BL/6 mice (8–10 weeks) were infected with JEV-P3 at 5 × 10^6^ plaque-forming units (PFUs) in 20 μl phosphate-buffered saline (PBS) *via* footpad injection. The data were analyzed and shown as Kaplan–Meier survival curves. **(B)** The mean behavior score of each mouse measured at 8:00–9:00 and 16:00–17:00 was calculated and analyzed. Data are shown as the mean ± SEM of all mice in each group. **(C)** The mean weight of each mouse at 8:00–9:00 and 16:00–17:00 was calculated and analyzed. Data are shown as the mean ± SEM of all mice in each group. **(D)** RIPK3^–/–^ (*n* = 9) and WT (*n* = 13) C57BL/6 mice (8–10 weeks) were infected with JEV-P3 at 100 PFU in 2 μl PBS *via* intracerebral injection. The death cases of each group were recorded every day, and then the data were analyzed and shown as Kaplan–Meier survival curves.

In our previous study, MLKL^–/–^ mice showed alleviated JE progression compared to WT mice to some extent. RIPK3, as the upstream signaling molecule of MLKL phosphorylation in classical necroptosis, has more complicated roles in apoptosis, inflammation, cytokine, and IFN production and the immunometabolic state (He and Wang, 2018). To determine the role of RIPK3 in JEV infection, RIPK3^–/–^ mice were infected with JEV by footpad injection and monitored daily for survival, weight, and behavioral score. The results showed that RIPK3^–/–^ mice had an increased survival rate compared with WT mice after JEV infection (Figure 1A). The average behavior score of RIPK3^–/–^ mice was lower than that of WT mice (Figure 1B). The weight in RIPK3^–/–^ mice was more stable (Figure 1C). Generally, RIPK3 deficiency led to decreased morbidity and mortality during JEV infection *in vivo*. In the early phase of infection, RIPK3^–/–^ mice showed more aggressive onset of JE than WT mice. We speculated that RIPK3^–/–^ monocytes and dendritic cells contributed to the propagation of JEV in the peripheral organs. Then, the RIPK3^–/–^ and WT mice were infected with JEV by intracerebral (IC) injection to avoid the peripheral immune system. The RIPK3^–/–^ mice were also more resistant to JEV infection than the WT mice (Figure 1D). Thus, the absence of RIPK3 in the CNS alleviated JE progression.

### Japanese Encephalitis Virus Infection Induced Receptor Interacting Serine/Threonine-Protein Kinase 3 Expression Which Contributed to Neuronal Death

**FIGURE 2:**
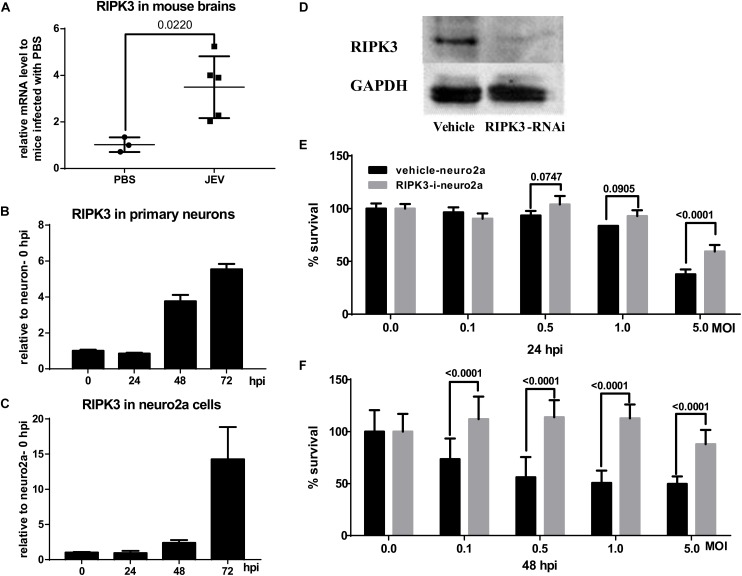
Japanese encephalitis virus (JEV) infection induced receptor interacting serine/threonine-protein kinase 3 (RIPK3) expression which contributed to neuronal death. **(A)** C57BL/6 mice were infected with JEV (*n* = 5) or PBS (*n* = 3) *via* footpad injection, and the brains were harvested. The expression of RIPK3 was evaluated by qPCR. The data represent the change relative to RIPK3 expression in phosphate-buffered saline (PBS)-treated mice. Data are shown as the mean ± SD. **(B)** The expression of RIPK3 in primary neurons at 24, 48, and 72 h post infection (hpi) after JEV infection was evaluated by qPCR. The data represent the change relative to the level in neurons at 0 hpi. Data are shown as the mean ± SD. Three independent experiments were performed. **(C)** The expression of RIPK3 in Neuro2a cells at 24, 48, and 72 hpi was tested by qPCR. The data represent the change relative to the level in neuro2a cells at 0 hpi. Data are shown as the mean ± SD. Three independent experiments were performed. **(D)** RIPK3 knockdown neuro2a cells were constructed by RIPK3-specific RNA interference mediated by a lentiviral vector, and positive cells were purified by puromycin selection. The expression of RIPK3 in RIPK3-RNAi-neuro2a cells and vehicle-neuro2a cells was detected by Western blotting (WB). The level of RIPK3 in RIPK3-RNAi-neuro2a cells decreased significantly. **(E,F)** The effect of RIPK3 on the viability of neuro2a cells during JEV infection. The survival rates of RIPK3-RNAi-neuro2a cells and vehicle-neuro2a cells after JEV infection at multiplicities of infection (MOIs) of 0.1, 0.5, 1.0, and 5 with six replicates were tested by cell viability assay kits at 24 and 48 hpi. The survival rate of RIPK3-RNAi-neuro2a cells was increased relative to that of vehicle-neuro2a cells after JEV infection, especially at 48 hpi. Data are presented as the mean ± SEM.

To explore the changes in RIPK3 during JEV infection *in vivo* and *in vitro*, the expression of RIPK3 was detected. After JEV infection, the expression of RIPK3 was increased in the CNS (Figure 2A). Moreover, the expression of RIPK3 was also increased in neurons and neuro2a cells following JEV infection (Figures 2B,C). The phosphorylation of RIPK3 led to classical MLKL-mediated necroptosis. In the JEV-infected mouse brains, PI-labeled necrotic cells were found to have increased expression of RIPK3 (Supplementary Figure S1). To identify the role of RIPK3 in neuronal survival, RIPK3-RNAi-neuro2a cells were constructed (Figure 2D). Knockdown of RIPK3 increased the survival rate of neuro2a cells after JEV infection with different PFUs and infection times (Figures 2E,F). Thus, the expression of RIPK3 in neuro2a cells contributed to JEV-induced neuronal death.

### Viral Loads Were Lower in the Brains of Receptor Interacting Serine/Threonine-Protein Kinase 3-Knockout Mice After Japanese Encephalitis Virus Infection *via* Intracerebral Injection

**FIGURE 3:**
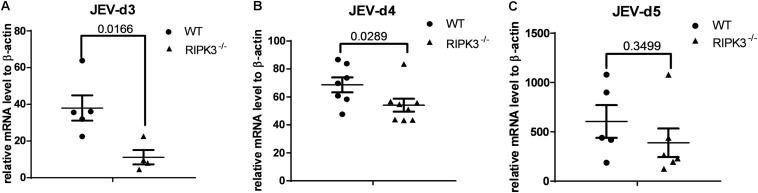
The Japanese encephalitis virus (JEV) load was lower in receptor interacting serine/threonine-protein kinase 3 (RIPK3)-knockout mice after JEV infection *via* intracerebral (IC) injection. RIPK3^–/–^ and wild-type (WT) C57BL/6 mice (8–10 weeks) were infected with 100 plaque-forming units (PFUs) JEV-P3 in 2 μl phosphate-buffered saline (PBS) *via* intracerebral injection. Mice were sacrificed, and the brains of each group were harvested at 3, 4, and 5 days post infection (dpi). The viral load in the brains was tested by qPCR. Data are presented as the mean ± SD. **(A)** The viral load in the brains of RIPK3^–/–^ (*n* = 4) mice and WT (*n* = 5) C57BL/6 mice at 3 dpi. **(B)** The viral load in the brains of RIPK3^–/–^ (*n* = 8) and WT (*n* = 7) C57BL/6 mice at 4 dpi. **(C)** The viral load in the brains of RIPK3^–/–^ (*n* = 6) and WT (*n* = 5) C57BL/6 mice at 5 dpi.

RIPK3 participated in the regulation of inflammation and cell survival, which directly or indirectly affected the propagation of virus during virus infection. To determine the role of RIPK3 in JEV propagation in the CNS, we tested the viral loads in the brains after JEV infection *via* IC injection at 3, 4, and 5 days. Surprisingly, the JEV RNA copy number in RIPK3^–/–^ mice was significantly less than that in WT mice at 3 and 4 dpi (Figures 3A,B). At 5 dpi, the viral load in most of the RIPK3^–/–^ mice was still lower than that in the WT mice (Figure 3C). This result was different from the infections with ZIKV and WNV, in which the viral load was increased in the CNS of RIPK3^–/–^ mice after virus infection because of the changed immunometabolism or decreased expression of chemokines in the neurons (Daniels et al., 2017, 2019).

### Receptor Interacting Serine/Threonine-Protein Kinase 3 (RIPK3) Promoted the Propagation of Japanese Encephalitis Virus in Neurons

**FIGURE 4:**
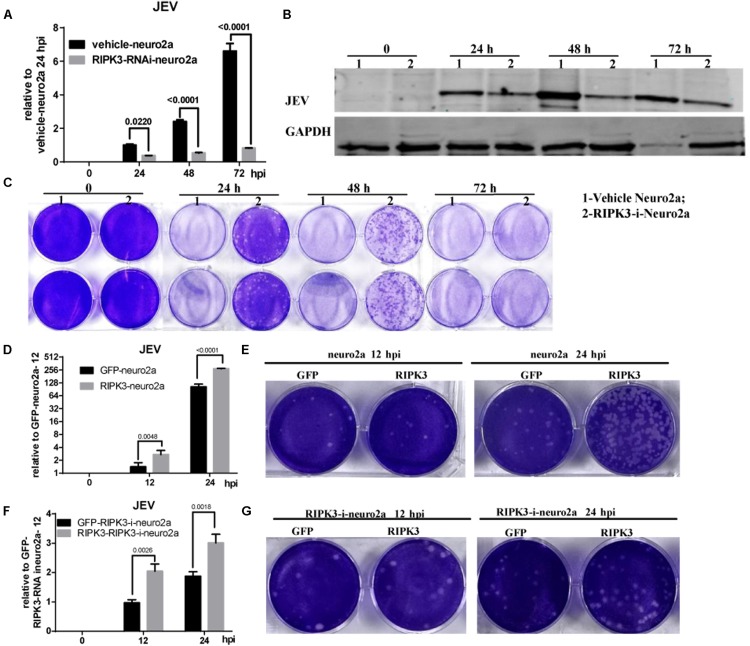
Receptor interacting serine/threonine-protein kinase 3 (RIPK3) promoted the propagation of Japanese encephalitis virus in neurons. **(A)** Vehicle-neuro2a cells and RIPK3-RNAi-neuro2a cells were infected with JEV-p3 at a multiplicity of infection (MOI) of 0.1 and collected at 24, 48, 72 h post infection (hpi) for RNA extraction. The expression of JEV was evaluated by qPCR. The data represent the change relative to the level in vehicle-neuro2a cells at 24 hpi. Data are presented as the mean ± SD. The experiments were repeated three times. **(B)** Protein from vehicle-neuro2a cells and RIPK3-RNAi-neuro2a cells was extracted at 24, 48, and 72 h after JEV infection, and the E protein of JEV was detected by Western blotting (WB). Representative images from three independent experiments are shown. **(C)** The supernatant from vehicle-neuro2a cells and RIPK3-RNAi-neuro2a cells was collected at 24, 48, and 72 h post JEV infection. The infectious JEV particles in the supernatant were detected by plaque assay with double wells at a dilution of 1:100. Representative images from three independent experiments are shown. **(D)** Neuro2a cells were transfected with pCMV-GFPSpark or pCMV-RIPK3-OFPSpark, and then GFP-neuro2a cells and RIPK3-neuro2a cells were infected with JEV-p3 at an MOI of 0.1 and collected at 12 and 24 hpi for RNA extraction. The expression of JEV was evaluated by qPCR. The data represent the change relative to the level in GFP-neuro2a 12 hpi. Data are shown as the mean ± SEM of three independent experiments. **(E)** The supernatant from GFP-neuro2a cells and RIPK3-neuro2a cells was collected at 12 and 24 h after JEV infection. The infectious JEV particles in the supernatant were detected by plaque assay at a dilution of 1:1,000. Representative images from three independent experiments are shown. **(F)** RIPK3-RNAi-Neuro2a cells were transfected with pCMV-GFPSpark or pCMV-RIPK3-OFPSpark, and then GFP-RIPK3-i-neuro2a cells and RIPK3-RIPK3-i-neuro2a cells were infected with JEV-p3 at an MOI of 0.1 and collected at 12 and 24 hpi for RNA extraction. The expression of JEV was evaluated by qPCR. The data represent the change relative to that in GFP-RIPK3-i-neuro2a cells at 12 hpi. Data are shown as the mean ± SEM of three independent experiments. **(G)** The supernatant from GFP-RIPK3-i-neuro2a cells and RIPK3-RIPK3-i-neuro2a cells was collected at 12 and 24 h post JEV infection. The infectious JEV particles in the supernatant were detected by plaque assay at a dilution of 1:100. Representative images from three independent experiments are shown.

Neurons are the main target cells of JEV infection in the CNS. To observe the effect of RIPK3 on JEV propagation in neurons, we infected neuro2a cells and RIPK3-RNAi-neuro2a cells with JEV at an MOI of 0.1 and detected the viral load using qPCR and WB. The mRNA levels of JEV decreased significantly in RIPK3-RNAi-neuro2a cells compared to vehicle neuro2a cells at different times of infection (Figure 4A), which was consistent with the JEV-E protein levels (Figure 4B). Then, the viral particles in the supernatants from different infection groups were assessed by plaque assay at a dilution of 1:100 (Figure 4C). There were many more infectious JEV particles in the supernatant from vehicle neuro2a cells than in the supernatant from RIPK3-RNAi-neuro2a cells. The results were further confirmed in primary neurons isolated from RIPK3^–/–^ and WT prenatal mice. The viral RNA levels (Supplementary Figure S2A), viral protein levels (Supplementary Figure S2B), and number of particles in the supernatant (Supplementary Figure S2C) from the RIPK3^–/–^ neurons were decreased compared with those from WT neurons. Thus, the propagation of JEV in RIPK3-deleted neurons was inhibited. Furthermore, to identify the role of RIPK3 in JEV replication, transient overexpression of RIPK3 in neuro2a cells and RIPK3-RNAi-neuro2a cells was conducted. The expression of RIPK3 in neuro2a cells was increased (Supplementary Figure S3A), and the cells were infected with JEV at 24 h after plasmid transduction. The viral copy number was increased in the RIPK3-overexpressing neuro2a cells (Figure 4D) as well as the infectious viral particles in the supernatant, as determined by plaque assay (Figure 4E) at 12 and 24 hpi. Moreover, RIPK3 supplementation in RIPK3-RNAi-neuro2a cells was performed (Supplementary Figure S3B), and viral copy numbers (Figure 4F) and infectious particles (Figure 4G) were also increased in RIPK3-RNAi-neuro2a cells complemented with RIPK3. In total, RIPK3 promoted the propagation of JEV in neuro2a cells.

### Receptor Interacting Serine/Threonine-Protein Kinase 3 Knockdown Had a Limited Effect on Japanese Encephalitis Virus Replication but Inhibited the Activation of Microglia

**FIGURE 5:**
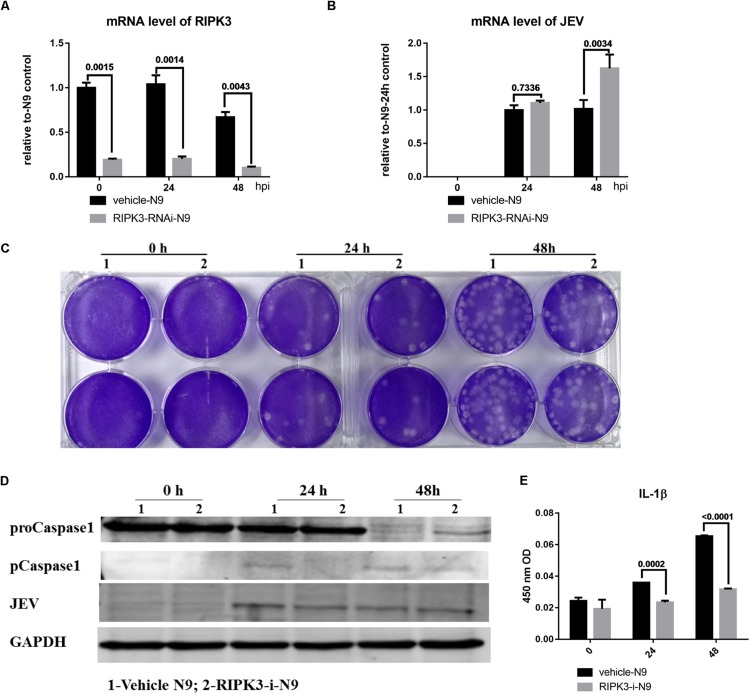
Receptor interacting serine/threonine-protein kinase 3 (RIPK3) knockdown had a limited effect on the level of Japanese encephalitis virus (JEV) in microglia. To explore whether RIPK3 knockout affected the level of JEV in microglia, RIPK3-RNAi-N9 cells were constructed and were infected with JEV at a multiplicity of infection (MOI) of 1. **(A)** The expression of RIPK3 in vehicle-N9 and RIPK3-RNAi-N9 cells was evaluated by qPCR. The expression of RIPK3 was decreased significantly in RIPK3-RNAi-N9 cells. **(B)**. RNA from vehicle-N9 and RIPK3-RNAi-N9 cells was extracted at 24 and 48 h after JEV infection. The JEV level was evaluated by qPCR. Data are presented as the mean ± SD. The experiments were repeated three times. **(C)** The supernatant from vehicle-N9 and RIPK3-RNAi-N9 cells was collected at 24 and 48 h post JEV infection. The infectious JEV particles in the supernatant were detected by plaque assay with double wells at a dilution of 1:100. Representative images from three independent experiments are shown. **(D)** Protein from vehicle-N9 and RIPK3-RNAi-N9 cells was extracted at 24 and 48 h after JEV infection. The JEV E protein, proCaspase1, and pCaspase1 were detected by Western blotting (WB). Representative images from three independent experiments are shown. **(E)**. The level of interleukin (IL)-1β in the supernatant from RIPK3-RNAi-N9 cells and vehicle-N9 cells was detected by ELISA. Data are presented as the mean ± SEM of three independent experiments.

Microglia, as the main resident immune defensive cells in the CNS, play important roles during JEV infection (Thongtan et al., 2012). After being exposed to JEV, microglia can be activated as innate immune cells and release a series of cytokines to recruit immune cells that contribute to immune defense as well as neuroinflammation. To explore whether knockdown of RIPK3 affected the level of JEV replication in microglia, RIPK3-RNAi-N9 cells were constructed. The expression of RIPK3 was decreased significantly in RIPK3-RNAi-N9 cells compared to the vehicle control cells (Figure 5A). Then, the viral load was detected at 24 and 48 h after JEV infection by qPCR and WB (Figures 5B,D). There was no significant difference in viral expression between RIPK3-RNAi-N9 and vehicle-N9 cells at 24 h. However, the expression of JEV RNA and protein was increased slightly in RIPK3-RNAi-N9 cells at 48 h. The amount of infectious JEV particles in the supernatant of RIPK3-RNAi-N9 cells was comparable to that of vehicle N9 cells (Figure 5C). Thus, RIPK3 had little effect on the propagation of JEV in N9 cells. Furthermore, the level of activated caspase-1 (Figure 5D) and the production of IL-1β after JEV infection (Figure 5E) in RIPK3-RNAi-N9 cells were demonstrated to decrease. Thus, the activation of microglia during JEV infection was inhibited in the absence of RIPK3, which was consistent with reports that RIPK3 participated in the formation of the inflammasome in microglia (Lawlor et al., 2015).

### Interferon (IFN)-Stimulated Genes, Especially IFN-Induced Protein 44-Like Gene, Were Upregulated in RIPK3^–/–^ Mouse Brains and Neurons After Japanese Encephalitis Virus Infection

**FIGURE 6:**
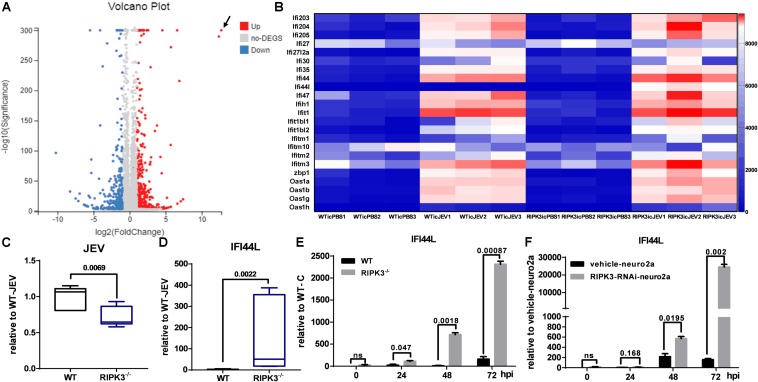
Interferon (IFN)-stimulated genes (ISGs), especially IFN-induced protein 44-like gene (IFI44L), were upregulated in receptor interacting serine/threonine-protein kinase 3-knockout (RIPK3^–/–^) mouse brains and neurons after Japanese encephalitis virus (JEV) infection. Wild-type (WT) and RIPK3^–/–^ mice (8–10 weeks) were injected intracerebrally with phosphate-buffered saline (PBS) or JEV 100 plaque-forming units (PFUs) in 2 μl PBS. Brains were harvested at 3 days post infection (dpi), and total RNA was extracted for RNA-sequencing. **(A)** The analysis of volcano plots of differentially expressed genes in the brains of WT and RIPK3^–/–^ mice after JEV infection. **(B)** The change in a series of ISGs between WT and RIPK3^–/–^ brains after JEV infection was analyzed according to RNA sequencing. In addition to IFI44L, the expression of oas1h, ifi1, zbp1, etc. was increased to some extent in RIPK3^–/–^ mouse brains after JEV infection. **(C)** WT (*n* = 3) and RIPK3^–/–^ (*n* = 3) mice were infected with JEV by intracerebral (IC) injection again, brains were collected at 3 dpi, and the viral load was evaluated by qPCR. Data are presented as the mean ± SD. **(D)**. The level of IFI44L in the brains of WT and RIPK3^–/–^ mice was detected by qPCR. Data are presented as the mean ± SD. **(E)** WT and RIPK3^–/–^ mouse-derived primary neurons were infected with JEV at a multiplicity of infection (MOI) of 0.1, and the expression of IFI44L was detected at 24, 48, and 72 hpi by qPCR. Data are presented as the mean ± SD. The experiments were repeated three times. **(F)** RIPK3-RNAi-neuro2a cells and vehicle-neuro2a cells were infected with JEV at an MOI of 0.1, and the expression of IFI44L was tested at 24, 48, and 72 hpi by qPCR. Data are presented as the mean ± SD. The experiments were repeated three times.

In contrast with previous reports that RIPK3 mediated the suppression of viruses in the CNS and neurons, the propagation of JEV in the CNS of RIPK3^–/–^ mice and RIPK3^–/–^ neurons was inhibited. To explore the mechanism involved, RNA-sequencing of brain tissues from RIPK3^–/–^ mice and WT mice treated with JEV or PBS *via* IC injection at 3 dpi was performed. According to the volcano plots of differentially expressed genes, ifi44l was the most significantly upregulated gene in RIPK3^–/–^ mouse brains compared to WT mouse brains after JEV infection (Figure 6A). Moreover, a number of ISGs in the brains also increased between RIPK3^–/–^ and WT mice after JEV infection and were more significant in RIPK3^–/–^ mice (Figure 6B). To clarify the expression of IFI44L mRNA, WT and RIPK3^–/–^ mice were injected with JEV *via* IC injection again. Brains were harvested at 3 dpi, and the levels of JEV RNA and IFI44L mRNA were evaluated by qPCR. Consistent with the above results, the level of JEV was relatively lower in RIPK3^–/–^ mice than in WT mice (Figure 6C), and the mRNA level of IFI44L increased significantly in RIPK3^–/–^ mice compared with WT mice (Figure 6D). Furthermore, the expression of IFI44L in WT and RIPK3^–/–^ primary neurons was detected by qPCR. The level of IFI44L increased significantly in RIPK3^–/–^ neurons after JEV infection (Figure 6E) as well as in RIPK3 knockdown neuro2a cells (Figure 6F). Thus, the absence of RIPK3 promoted the expression of IFI44L in neurons and inhibited viral replication during JEV infection.

### The Increase of Interferon-Induced Protein 44-Like Gene Was Independent of the Phosphorylation of Receptor Interacting Serine/Threonine-Protein Kinase 3 or Mixed Lineage Kinase Domain-Like Pseudokinase

**FIGURE 7:**
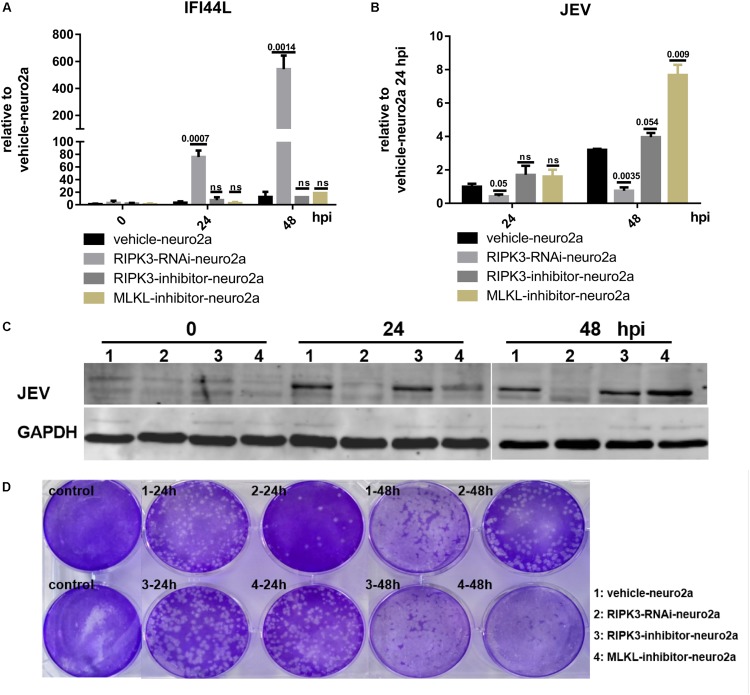
The increase in interferon-induced protein 44-like gene (IFI44L) was independent of the phosphorylation of receptor interacting serine/threonine-protein kinase 3 (RIPK3) or mixed lineage kinase domain-like pseudokinase (MLKL). The phosphorylation of RIPK3 and subsequently MLKL formed the classical signal of necroptosis. To explore whether the inhibition of ifi44l was dependent on the phosphorylation of RIPK3 or MLKL, Neuro2a cells were treated with 1.5 nM RIPK3 kinase inhibitor GSK872 (RD, United States) or 1 μM MLKL inhibitor necrosulfonamide (RD, United States) 2 h before Japanese encephalitis virus (JEV) infection, and the inhibitors remained until 48 h post infection (hpi). The experiments were repeated three times. **(A)** RNA from vehicle-neuro2a cells, RIPK3-RNAi-neuro2a cells, and inhibitor-treated neuro2a cells was extracted at 24 and 48 hpi, and the expression of IFI44L was evaluated by qPCR. Data are presented as the mean ± SD. **(B)** The viral load in vehicle-neuro2a cells, RIPK3-RNAi-neuro2a cells, and inhibitor-treated neuro2a cells was detected by qPCR. Data are presented as the mean ± SD. **(C)** Protein from RIPK3-RNAi-neuro2a cells, vehicle-neuro2a cells, and inhibitor-treated neuro2a cells was extracted, and the JEV E protein was tested by Western blotting (WB). **(D)** Supernatants from RIPK3-RNAi-neuro2a cells, vehicle-neuro2a cells, and inhibitor-treated neuro2a cells were collected after JEV infection for 24 and 48 h. The infectious JEV particles in the supernatant were detected by plaque assay at a dilution of 1:100.

The phosphorylation of RIPK3 and subsequent MLKL activation are key to the classical necroptosis pathway (Weinlich et al., 2017). To explore whether the inhibition of IFI44L was dependent on the phosphorylation of RIPK3 or MLKL, Neuro2a cells were treated with inhibitors of RIPK3 or MLKL phosphorylation (Supplementary Figure S4). The expression of IFI44L was tested by qPCR at 24 and 48 hpi. The level of IFI44L mRNA increased significantly in RIPK3-RNAi-neuro2a cells but not in inhibitor-treated groups (Figure 7A). Furthermore, the viral loads tested by qPCR and WB were decreased in RIPK3-RNAi-neuro2a cells (Figures 7B,C), while virus replication was increased slightly after treatment with inhibitors, especially in pMLKL inhibitor-treated neuro2a cells, at 48 h. Moreover, the infectious viral particles in the RIPK3-RNAi-neuro2a cells were decreased but not in the neuro2a cells treated with inhibitors (Figure 7D). Thus, the inhibition of JEV replication in RIPK3-RNAi-neuro2a cells did not rely on the phosphorylation of RIPK3 or MLKL.

### Interferon-Induced Protein 44-Like Gene (IFI44L) Inhibited Japanese Encephalitis Virus Propagation in RIPK3-RNAi Neuro2a Cells

**FIGURE 8:**
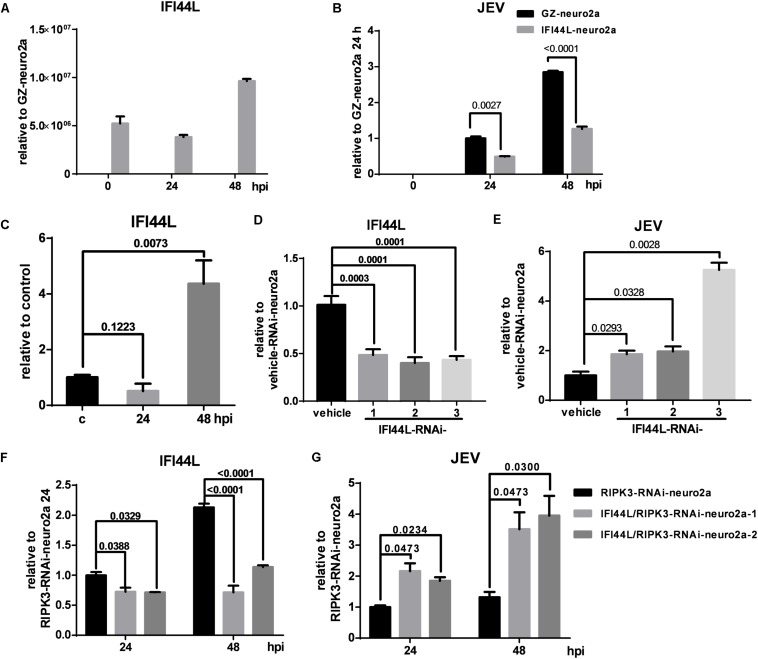
The propagation of Japanese encephalitis virus (JEV) was inhibited in interferon-induced protein 44-like gene (IFI44L)-overexpressing neuro2a cells and enhanced in IFI44L- and receptor interacting serine/threonine-protein kinase 3 (RIPK3) double knockdown neuro2a cells. **(A)** To identify the effect of IFI44L on JEV propagation, IFI44L-overexpressing neuro2a cells (IFI44L-neuro2a) were constructed, and GFP-Zeocin-overexpressing neuro2a cells (GZ-neuro2a) were constructed as a control. The experiments were repeated three times. The expression of IFI44L was tested by qPCR. Data are presented as the mean ± SD. **(B)** IFI44L-neuro2a cells and GZ-neuro2a cells were infected with JEV at a multiplicity of infection (MOI) of 0.1, and the virus load was detected by qPCR at 24 and 48 h post infection (hpi). **(C)** The expression of IFI44L in neuro2a cells after JEV infection for 24 and 48 h was tested by qPCR. **(D,E)** Neuro2a cells were treated with three different IFI44L-interfering lentiviruses targeting different segments of IFI44L. The expression of IFI44L and viral mRNA was tested by qPCR at 48 hpi. Data are presented as the mean ± SD. The experiments were repeated three times. **(D)** The expression of IFI44L was decreased in IFI44L-RNAi-neuro2a cells compared to vehicle-neuro2a cells. **(E)** The virus load in IFI44L-RNAi-neuro2a cells was higher than that in vehicle-neuro2a cells. **(F,G)**. To identify the role of IFI44L in JEV propagation in RIPK3-RNAi-neuro2a cells, IFI44L knockdown was performed in RIPK3-RNAi-neuro2a cells. The expression of IFI44L **(F)** and viral mRNA **(G)** in RIPK3-RNAi-neuro2a cells and IFI44L/RIPK3-RNAi-neuro2a cells was tested by qPCR at 24 and 48 hpi. Data are presented as the mean ± SD. The experiments were repeated three times.

To identify the effect of IFI44L on JEV propagation, IFI44L-overexpressing neuro2a cells (IFI44L-neuro2a) were constructed. The mRNA level of IFI44L increased significantly in IFI44L-neuro2a cells (Figure 8A). The viral RNA level decreased significantly in IFI44L-neuro2a cells compared with GZ-neuro2a cells after JEV infection at 24 and 48 h (Figure 8B). The expression of IFI44L was slightly increased in neuro2a cells at 48 hpi (Figure 8C). Then, IFI44L was downregulated in neuro2a cells at 48 hpi using three IFI44L-targeting shRNAs (Figure 8D and Supplementary Figure S5A). Viral RNA copy numbers increased in IFI44L-RNAi neuro2a cells compared to vehicle neuro2a cells (Figure 8E). This result indicated that IFI44L in neuro2a cells inhibited JEV propagation. Furthermore, IFI44L in RIPK3-i-neuro2a cells was downregulated *via* shRNAs (Figure 8F and Supplementary Figure S5B). The viral RNA level increased in IFI44L/RIPK3 double knockdown neuro2a cells compared with RIPK3-RNAi-neuro2a cells after JEV infection (Figure 8G). Thus, the upregulation of IFI44L in RIPK3-RNAi-neuro2a cells contributed to the inhibition of JEV propagation.

## Discussion

Recently, a number of studies found that RIPK3 mediated complicated roles in cell death, inflammation, and immune defense during virus infection depending on different host cells and viruses (Orozco and Oberst, 2017). In this study, we found that RIPK3^–/–^ mice were more resistant to JEV infection during peripheral and intracerebral infection than WT mice. The expression of RIPK3 was increased in neuronal cells following JEV infection, and the increased RIPK3 promoted JEV propagation. Moreover, the viral load was decreased in RIPK3-deleted neuronal cells because of the increased expression of IFI44L. Thus, we speculated that the induced expression of RIPK3 in virus-infected neurons might be a strategy for JEV to evade cellular innate immunity.

The phosphorylation of RIPK1, RIPK3, and subsequently MLKL induces canonical necroptosis followed by DAMP production and inflammation (Pasparakis and Vandenabeele, 2015). In our previous study, we demonstrated that MLKL-mediated necroptosis accelerated JEV-induced neuroinflammation in mice and that MLKL^–/–^ mice showed alleviated JE progression. In this study, we found that morbidity and mortality were decreased in RIPK3^–/–^ mice compared to WT mice after peripheral JEV infection, and JE progression was alleviated in RIPK3^–/–^ mice after intracerebral infection. Thus, RIPK3 accelerated JE progression in mice. The expression of RIPK3 was increased in neurons after JEV infection. RIPK3-silenced neuro2a cells showed increased cell viability during JEV infection compared with vehicle neuro2a cells. Thus, RIPK3 promoted neuronal death during JEV infection.

It has been shown that RIPK3/MLKL-mediated necroptosis has antiviral function in fibroblast and epithelial cells during lytic virus infection by destroying viral reservoirs (Nogusa et al., 2016). Additionally, RIPK3 has been demonstrated to play complicated roles in virus propagation in cell death-independent ways. During IAV infection in macrophages, on the one hand, the virus induced RIPK3 accumulation in mitochondria and interfered with RIPK1/MAVS interactions to decrease IFN-β expression, which might be an immune evasion strategy adopted by IAV. On the other hand, the increased RIPK3 could activate protein kinase R (PKR), which stabilized IFN-β mRNA, leading to the increased protein level of IFN-β, which might be the response of the host cells to counteract viral evasion (Downey et al., 2017). During CVB infection in intestinal epithelial cells (IECs), RIPK3 promoted CVB infection *via* the positive regulation of autophagic flux (Harris et al., 2015). In neurons, the tug-of-war between cellular immune defense and viral evasion is more complex. Daniels et al. (2019) found that the activation of RIPK1 and RIPK3 in neurons induced the upregulation of IRG1 and the metabolite itaconate to restrict viral replication through an immune-metabolism mechanism during ZIKV infection. In our study, the propagation of JEV was inhibited in RIPK3-deleted neurons and was promoted in RIPK3-overexpressing neuro2a cells. The differences might be explained by the fact that RIPK3 exerts different functions depending on the virus and the host cell. Moreover, we speculated that the increased expression of RIPK3 following JEV infection might be a strategy for JEV to evade cellular innate immunity. The components of JEV particles will be explored in subsequent studies to identify the exact mechanism by which JEV infection promotes RIPK3 expression in neuronal cells.

ISGs are the cellular factors induced by type I IFN in host cells to suppress viral replication. Hundreds of ISGs have been identified, some of which are broad-spectrum antivirals, while others are specific for viruses and cells. Moreover, the antiviral activity of ISGs can be enhanced through synergistic effects (Schoggins, 2019). IFI44L has been found to inhibit the replication of HCV, ZIKV, and DENV. It has been reported that IFI44L inhibited the replication of HCV in Huh-7 cells (Schoggins et al., 2011). In addition, the low levels of IFI44L, IFI27, and STAT1 contributed to the high viral load because of the impaired IFN production caused by HCV NS3-4A protease in HCV patients (Bellecave et al., 2010). Recently, Robinson et al. (2018) also showed that the failure to induce IFI44L contributed to the long-term propagation of ZIKV in germ cells. The expression of ISGs, including IFI44L, OAS1, and IFIT3, was downregulated by the NS4B protein of dengue virus (DENV) in human cells and thus resulted in high viral replication, which was an immune evasion strategy for DENV (Bui et al., 2018). In this study, a series of ISGs were increased in RIPK3^–/–^ mouse brains after JEV infection, among which IFI44L was increased most significantly compared with that in the WT. The antiviral role of IFI44L in neuronal cells during JEV infection was demonstrated by both the overexpression and knockdown of IFI44L. However, IFI44L did not completely inhibit JEV replication in RIPK3^–/–^ neurons. This did not rule out the role of other molecules. In addition to IFI44L, other ISGs, such as ZBP1, OAS1, and Gbp2b, were also upregulated in RIPK3 knockout mice and neuronal cells and might defend against JEV synergistically.

Neurons were the main host cells of JEV propagation. The expression of IFI44L was higher in RIPK3^–/–^ neurons during JEV infection. However, the relationship between IFI44L expression and RIPK3 was unclear. We further compared the levels of the main cytokines in WT and RIPK3^–/–^ primary neurons after JEV infection. The mRNA level of CXCL10 increased significantly in WT neurons upon JEV infection compared to RIPK3^–/–^ neurons, which was consistent with previous reports that the production of CXCL10 was impaired in RIPK3^–/–^ neurons during WNV infection (Supplementary Figure S6A). The level of tumor necrosis factor (TNF)α was also higher in WT neurons than RIPK3^–/–^ neurons after JEV infection, which might be the result of different viral loads (Supplementary Figure S6B). Then, we detected the levels of IFNs, including IFNα, IFNβ, and IFNγ. Compared to those in WT neurons without JEV infection, IFNs in both WT and RIPK3^–/–^ neurons increased after JEV stimulation (Supplementary Figures S6C–E). Overall, the total expression levels of IFNα in WT neurons were higher during JEV infection than those in RIPK3^–/–^ neurons, while the expression of IFNβ and IFNγ were comparable. In terms of relative changes, the increase in magnitude of IFNα relative to that in the corresponding control neurons was comparable between WT and RIPK3^–/–^ neurons, but the increase in magnitude of IFNβ and IFNγ was higher in RIPK3^–/–^ neurons (Supplementary Figures S6F–H). Taken together, these results indicated that the baseline IFN expression in RIPK3^–/–^ neurons was lower than that in WT neurons. Upon JEV stimulation, the higher increase in the magnitude of IFN in RIPK3^–/–^ neurons might partly contribute to the production of IFI44L. However, more studies are needed to explore the mechanism by which RIPK3 regulates ifi44l expression.

In summary, RIPK3 has complicated roles in neuroinflammation and virus propagation during viral infection. In our study, we found a novel role of RIPK3 in JEV propagation in neurons, which is different from the role of RIPK3 in CNS infected with WNV of the same genus *Flavivirus*. Our findings further reinforce the intricate and subtle nature of the game between host and virus. We believe that RIPK3 may be a new therapeutic target for the development of virus replication inhibitors to treat JEV-induced encephalitis.

## Data Availability Statement

All datasets generated for this study are included in the article/Supplementary Material.

## Ethics Statement

The animal study was reviewed and approved by the Animal Care and Use Committee of the Laboratory Animal Center, Air Force Medical University. The number of Animal Experimental Ethical Inspection is 20160112.

## Author Contributions

PB contributed to the conception and design, data collection and assembly, data analysis and interpretation, and manuscript writing. CY contributed to the data collection and assembly. XZ contributed to the data analysis and manuscript writing. CL, JiaY, ML, YW, JinY, and YuZ contributed to the data collection. FZ, JL, and YiZ contributed to the administrative support and provision of study material. ZJ contributed to the conception and design, administrative support, and final approval of the manuscript. YL contributed to the conception and design, financial support, and manuscript writing.

## Conflict of Interest

The authors declare that the research was conducted in the absence of any commercial or financial relationships that could be construed as a potential conflict of interest.

## Footnotes

1 http://report.bgi.com

## Supplementary Material

FIGURE S1 The colocalization of PI and RIPK3 in the brains of JEV-infected mice. WT C57BL/6 mice infected with JEV via footpad injection were administered PI intraperitoneally at 5 dpi and euthanized 1 h later. The expression of RIPK3 (green) was detected, and the colocalization of RIPK3 and PI (red) was recorded. Cells with both RIPK3 and PI positivity (white row) and RIPK3 positivity without PI (blue row) were all detected.

FIGURE S2 The propagation of JEV was inhibited in RIPK3^–/–^ primary neurons. Primary neurons from WT and RIPK3^–/–^ mice were isolated and cultured for 1 week and then infected with JEV at an MOI of 0.1. Data are presented as the mean ± SD. The experiments were repeated three times. **(A)** RNA was extracted at 24, 48, and 72 h after JEV infection, and the level of JEV was detected by qPCR. The expression of JEV mRNA in each group was normalized to actin-β expression. Then, the relative fold change in each group was calculated based on the normalized mean expression of WT at 24 h. **(B)** Protein from WT and RIPK3^–/–^ neurons was extracted at 24, 48, and 72 h after JEV infection, and the E protein of JEV was tested by WB. **(C)** Supernatants from WT and RIPK3^–/–^ neurons were collected at 24, 48, and 72 h post JEV infection. The infectious JEV particles in the supernatant were detected by plaque assay with double wells at a dilution of 1:1000.

FIGURE S3 The expression of RIPK3 after pCMV-GFPSpark or pCMV-RIPK3-OFPSpark transfection. **(A)** Neuro2a cells were transfected with pCMV-GFPSpark or pCMV-RIPK3-OFPSpark, and then GFP-neuro2a cells and RIPK3-neuro2a cells were infected with JEV-p3 at an MOI of 0.1 and collected at 12 and 24 hpi for RNA extraction. The expression of RIPK3 was tested by qPCR. **(B)** RIPK3-RNAi-Neuro2a cells were transfected with pCMV-GFPSpark or pCMV-RIPK3-OFPSpark, and then GFP-RIPK3-i-neuro2a cells and RIPK3-RIPK3-i-neuro2a cells were infected with JEV-p3, MOI = 0.1 and collected at 12 and 24 hpi for RNA extraction. The expression of RIPK3 was tested by qPCR.

FIGURE S4 The expression of pRIPK3 and pMLKL in each group. Vehicle-neuro2a cells, RIPK3-RNAi-neuro2a cells, and inhibitor-treated neuro2a cells were collected for protein extraction at 48 hpi. The protein levels of pRIPK3 and pMLKL were detected by WB.

FIGURE S5 The expression of IFI44L in each group. **(A)** Neuro2a cells were treated with three different IFI44L interfering lentiviruses targeting different segments of IFI44L. The expression of IFI44L was evaluated by WB at 48 hpi. **(B)** To identify the role of IFI44L in JEV propagation in RIPK3-RNAi neuro2a cells, IFI44L knockdown was performed in RIPK3-RNAi-neuro2a cells. The expression of IFI44L in RIPK3-RNAi-neuro2a cells and IFI44L/RIPK3-RNAi-neuro2a cells was evaluated by WB at 24 and 48 hpi.

FIGURE S6 The expression of IFNs in primary neurons after JEV infection. WT and RIPK3^–/–^ mouse-derived primary neurons were infected with JEV; MOI = 0.1. RNA was extracted, and the expression of CXCL10, TNFα and IFNs was evaluated by qPCR at 24, 48, and 72 hpi. **(A)** The expression of CXCL10 in neurons was increased after JEV infection and was higher in WT neurons than RIPK3^–/–^ neurons. **(B)** The expression of TNFα in WT and RIPK3^–/–^ neurons. **(C–E)** Changes in the expression of IFNα, IFNβ and IFNγ in WT and RIPK3^–/–^ neurons after JEV infection relative to the WT control at 24, 48, and 72 hpi. **(F–H)** Changes in the expression of IFNα, IFNβ, and IFNγ in neurons after JEV infection relative to those in WT or RIPK3^–/–^ control neurons, respectively.

TABLE S1 shRNA targeting sequences, PCR primers and antibodies used in this study.
