# Supplementary figures and images for: RIPK3 Promotes JEV Replication in Neurons via Downregulation of IFI44L
Source: Front Microbiol. 2020 Mar 24;11:368. doi: 10.3389/fmicb.2020.00368 (PMC7105639; doi:10.3389/fmicb.2020.00368)

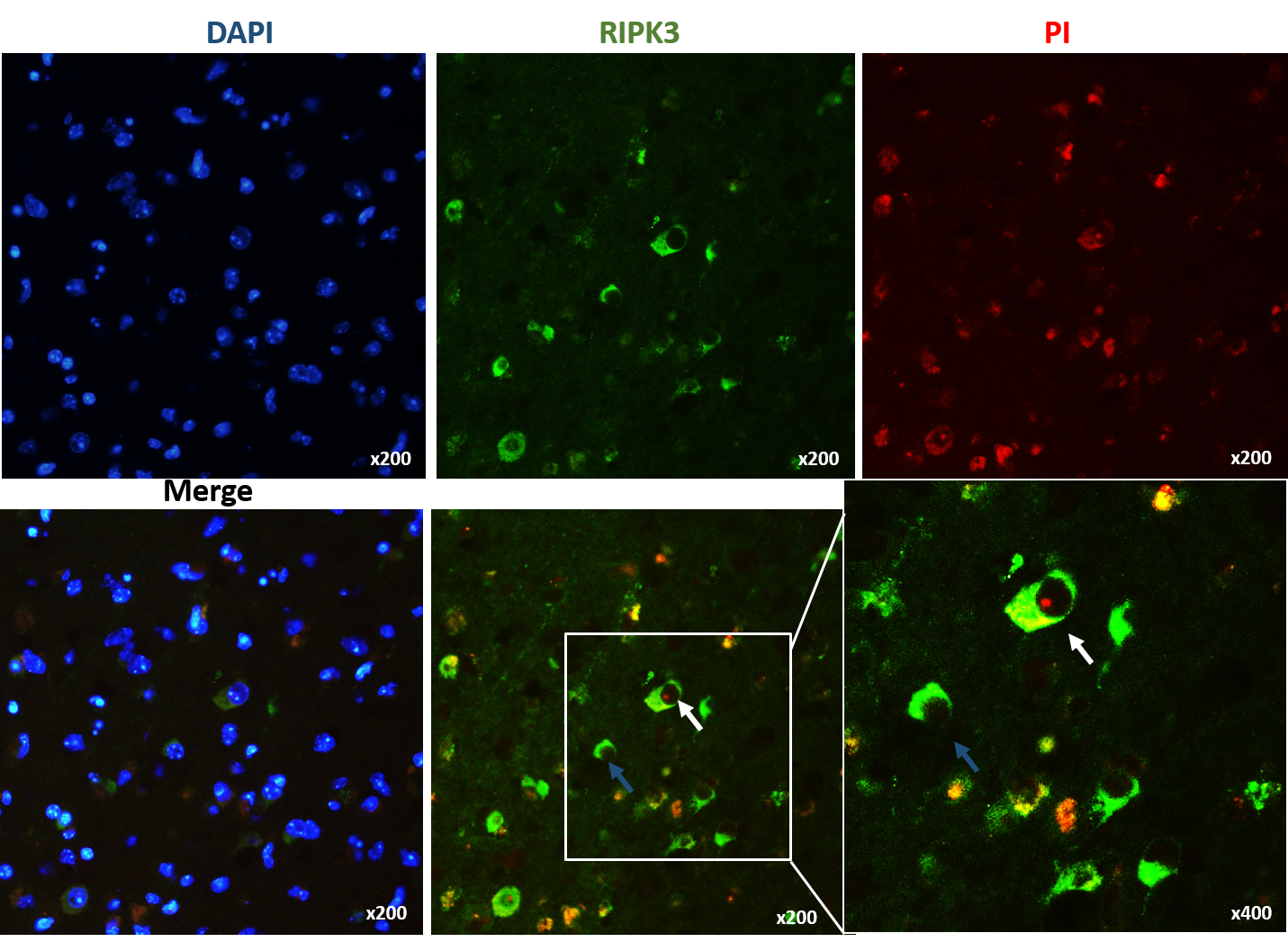

Supplement: FIGURE S1 — The colocalization of PI and RIPK3 in the brains of JEV-infected mice. WT C57BL/6 mice infected with JEV via footpad injection were administered PI intraperitoneally at 5 dpi and euthanized 1 h later. The expression of RIPK3 (green) was detected, and the colocalization of RIPK3 and PI (red) was recorded. Cells with both RIPK3 and PI positivity (white row) and RIPK3 positivity without PI (blue row) were all detected. [file Image_1.TIF]

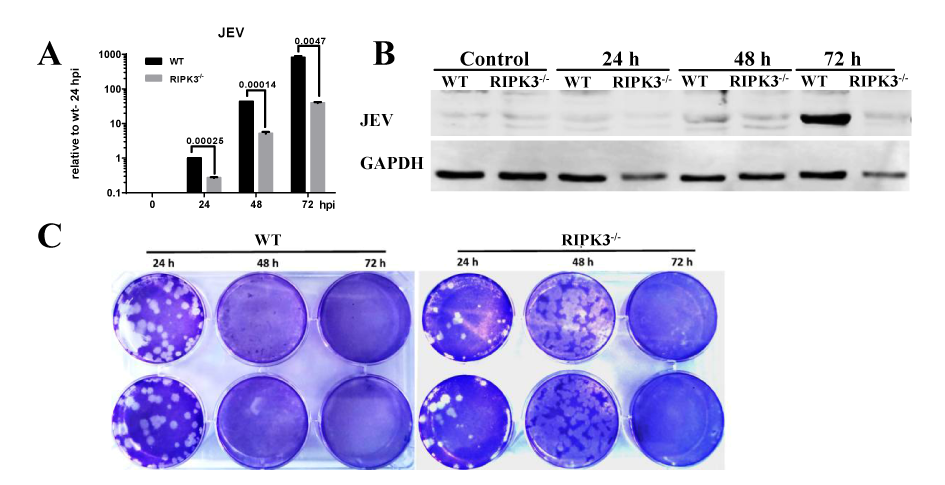

Supplement: FIGURE S2 — The propagation of JEV was inhibited in RIPK3–/– primary neurons. Primary neurons from WT and RIPK3–/– mice were isolated and cultured for 1 week and then infected with JEV at an MOI of 0.1. Data are presented as the mean ± SD. The experiments were repeated three times. (A) RNA was extracted at 24, 48, and 72 h after JEV infection, and the level of JEV was detected by qPCR. The expression of JEV mRNA in each group was normalized to actin-β expression. Then, the relative fold change in each group was calculated based on the normalized mean expression of WT at 24 h. (B) Protein from WT and RIPK3–/– neurons was extracted at 24, 48, and 72 h after JEV infection, and the E protein of JEV was tested by WB. (C) Supernatants from WT and RIPK3–/– neurons were collected at 24, 48, and 72 h post JEV infection. The infectious JEV particles in the supernatant were detected by plaque assay with double wells at a dilution of 1:1000. [file Image_2.TIF]

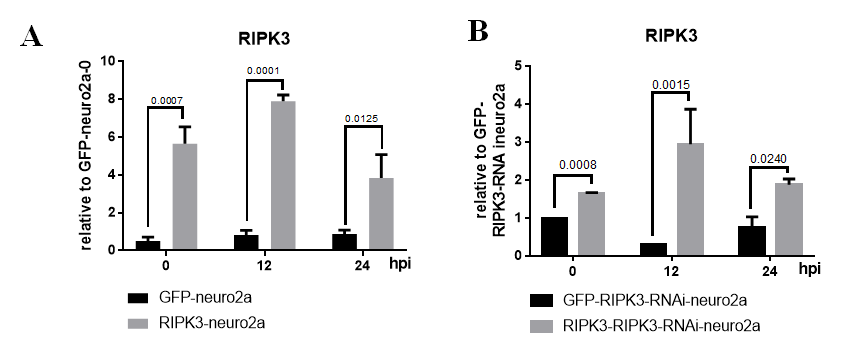

Supplement: FIGURE S3 — The expression of RIPK3 after pCMV-GFPSpark or pCMV-RIPK3-OFPSpark transfection. (A) Neuro2a cells were transfected with pCMV-GFPSpark or pCMV-RIPK3-OFPSpark, and then GFP-neuro2a cells and RIPK3-neuro2a cells were infected with JEV-p3 at an MOI of 0.1 and collected at 12 and 24 hpi for RNA extraction. The expression of RIPK3 was tested by qPCR. (B) RIPK3-RNAi-Neuro2a cells were transfected with pCMV-GFPSpark or pCMV-RIPK3-OFPSpark, and then GFP-RIPK3-i-neuro2a cells and RIPK3-RIPK3-i-neuro2a cells were infected with JEV-p3, MOI = 0.1 and collected at 12 and 24 hpi for RNA extraction. The expression of RIPK3 was tested by qPCR. [file Image_3.TIF]

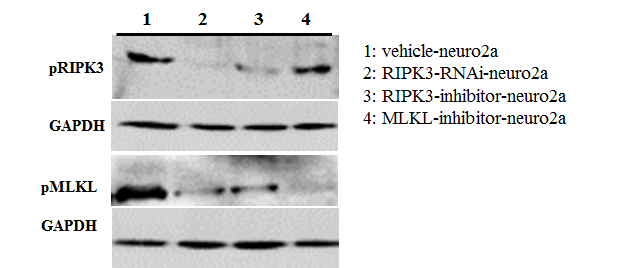

Supplement: FIGURE S4 — The expression of pRIPK3 and pMLKL in each group. Vehicle-neuro2a cells, RIPK3-RNAi-neuro2a cells, and inhibitor-treated neuro2a cells were collected for protein extraction at 48 hpi. The protein levels of pRIPK3 and pMLKL were detected by WB. [file Image_4.TIF]

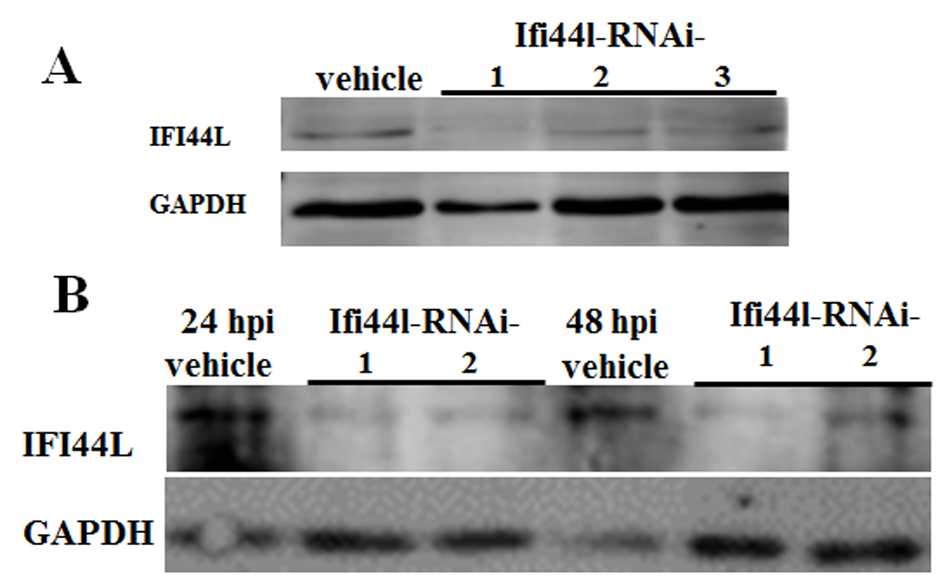

Supplement: FIGURE S5 — The expression of IFI44L in each group. (A) Neuro2a cells were treated with three different IFI44L interfering lentiviruses targeting different segments of IFI44L. The expression of IFI44L was evaluated by WB at 48 hpi. (B) To identify the role of IFI44L in JEV propagation in RIPK3-RNAi neuro2a cells, IFI44L knockdown was performed in RIPK3-RNAi-neuro2a cells. The expression of IFI44L in RIPK3-RNAi-neuro2a cells and IFI44L/RIPK3-RNAi-neuro2a cells was evaluated by WB at 24 and 48 hpi. [file Image_5.TIF]

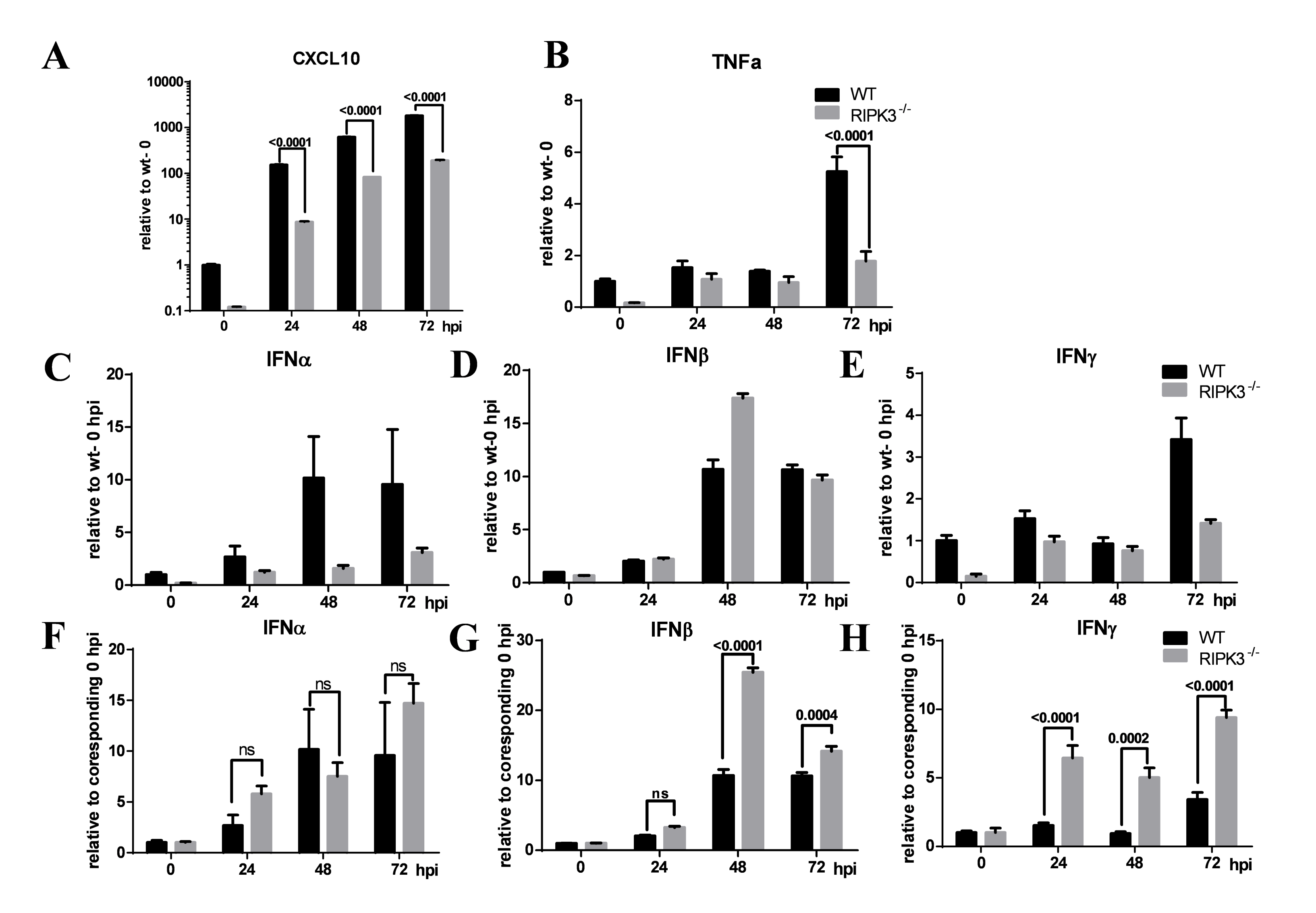

Supplement: FIGURE S6 — The expression of IFNs in primary neurons after JEV infection. WT and RIPK3–/– mouse-derived primary neurons were infected with JEV; MOI = 0.1. RNA was extracted, and the expression of CXCL10, TNFα and IFNs was evaluated by qPCR at 24, 48, and 72 hpi. (A) The expression of CXCL10 in neurons was increased after JEV infection and was higher in WT neurons than RIPK3–/– neurons. (B) The expression of TNFα in WT and RIPK3–/– neurons. (C–E) Changes in the expression of IFNα, IFNβ and IFNγ in WT and RIPK3–/– neurons after JEV infection relative to the WT control at 24, 48, and 72 hpi. (F–H) Changes in the expression of IFNα, IFNβ, and IFNγ in neurons after JEV infection relative to those in WT or RIPK3–/– control neurons, respectively. [file Image_6.TIF]
