# Supplementary material for: RIPK3 Promotes JEV Replication in Neurons via Downregulation of IFI44L
Source: Front Microbiol. 2020 Mar 24;11:368. doi: 10.3389/fmicb.2020.00368 (PMC7105639; doi:10.3389/fmicb.2020.00368)
Supplement: TABLE S1 — shRNA targeting sequences, PCR primers and antibodies used in this study. [file Table_1.DOCX]

**Sequence for shRNA**

**Primers for overexpression**

Ifi44l-F: 5’- GACGCAGATCTAGGAACATGAAAGTGACAGCCAGATTG -3’

Ifi44l-R: 5’- GACGCACGCGTGAAAGGAGCCAGGTTACCAGTATCCTCG-3’

| RIPK3 | 5’-GCTGGAGTTTGTGGGTAAAGG-3’ |
| --- | --- |
| IFI44L-CDS1 | 5’- TATCAGTCCAGTGCTCATAAA-3’ |
| IFI44L-CDS2 | 5’ -GGTTTCAGAAGGTTCGTATTC-3’ |
| IFI44L-CDS3: | 5’-GACTCTGAAGAGCTGATAATT-3’ |

**Primers for qPCR**

| JEV | F: AGACAAGCAGATCAACCACCATT R: CCCTCCAATAGAGCCAAAGTCC |
| --- | --- |
| TNF-α | F: CTG AAC TTC GGG GTG ATC GGT R: ACG TGG GCT ACA GGC TTG TCA |
| IFN-γ | F: GGCCATCAGCAACATAAGCGT R: TGGGTTGTTGACCTCAAACTTGGC |
| IFN-α | F: TCC TGA ACC TCT TCA CAT CAA A R: ACA GGC TTG CAG GTC ATT GAG |
| IFN-β | F: CTCCACCACAGCCCTCTC R: CATCTTCTCCGTCATCTCCATAG |
| IL-1β | F: GCAACTGTTCCTGAACTCAACT R: ATCTTTTGGGGTCCGTCAACT |
| IFI44L | F: TTCAACTCAGTGGAAGTCTGCT R: GGAGTGTTTCCCCGCTTTTTC |
| CXCL10 | F: CAT TCT TTT TCA TCG TGG CA R: AAG TGC TGC CGT CAT TTT CT |
| RIPK3 | F: CTCCGTGCCTTGACCTACTG R: AACCATAGCCTTCACCTCCC |
| β-Actin | F: TGACGGGGTCACCCACACTG R: AAGCTGTAGCCGCGCTCGGT |
